# Ribosomal protein NtRPL17 interacts with kinesin-12 family protein NtKRP and functions in the regulation of embryo/seed size and radicle growth

**DOI:** 10.1093/jxb/erx361

**Published:** 2017-10-14

**Authors:** Shujuan Tian, Jingjing Wu, Yuan Liu, Xiaorong Huang, Fen Li, Zhaodan Wang, Meng-Xiang Sun

**Affiliations:** 1 State Key Laboratory of Hybrid Rice, College of Life Sciences, Wuhan University, Wuhan, China; 2 College of Life Sciences, Henan Normal University, Xinxiang, China

**Keywords:** Cell division, embryogenesis, G2/M transition, kinesin, ribosomal protein, seed size

## Abstract

We previously reported that a novel motor protein belonging to the kinesin-12 family, NtKRP, displays critical roles in regulating embryo and seed size establishment. However, it remains unknown exactly how NtKRP contributes to this developmental process. Here, we report that a 60S ribosomal protein NtRPL17 directly interacts with NtKRP. The phenotypes of *NtRPL17* RNAi lines show notable embryo and seed size reduction. Structural observations of the *NtRPL17*-silenced embryos/seeds reveal that the embryo size reduction is due to a decrease in cell number. In these embryos, cell division cycle progression is delayed at the G2/M transition. These phenotypes are similar to that in *NtKRP*-silenced embryos/seeds, indicating that NtKRP and NtRPL17 function as partners in the same regulatory pathway during seed development and specifically regulate cell cycle progression to control embryo/seed size. This work reveals that NtRPL17, as a widely distributed ribosomal protein, plays a critical role in seed development and provides a new clue in the regulation of seed size. Confirmation of the interaction between NtKRP and NtRPL17 and their co-function in the control of the cell cycle also suggests that the mechanism might be conserved in both plants and animals.

## Teaser

Direct interaction between NtRPL17 and the kinesin NtKRP controls cell cycle progression in plants and reveals a novel regulatory role for ribosomal proteins in seed size establishment.

## Introduction

Seed development is critical for plant reproduction, especially for crop yield. Consequently, the mechanism regulating seed development has drawn great attention for decades. One of the essential processes in seed development is seed size control. As seed size is one of the most important yield traits, study of the molecular mechanisms controlling seed size is an attractive topic for plant scientists (Orozco-Arroyo *et al.*, 2015).

In recent decades, many key regulators for seed size control have been identified in plants. In rice, brassinosteroid (BR) biosynthesis-defective dwarf mutants, namely *dwarf1* (*d1*), *dwarf2* (*d2*), *dwarf11* (*d11*), BR-deficient *dwarf1* and BR-deficient *dwarf2* share a similar seed phenotype, producing grains with significantly reduced seed length (Hong *et al.*, 2005; Tanabe *et al.*, 2005). In Arabidopsis, an *auxin response factor 2* (*arf2*)/megaintegumenta (*mnt*) mutant displays increased seed size and weight because of additional cell division in the integuments, causing enlarged seed coats (Schruff *et al.*, 2006). A triple mutant of the cytokinin receptor genes *ARABIDOPSIS HISTIDINE KINASE 2* (*AHK2*), *AHK3*, and *AHK4* produces seeds that are more than twice the size of those from wild-type plants, which is mainly due to an increase in the corresponding embryo size (Riefler *et al.*, 2006). *MINI SEED3* (*MINI3*), a WRKY family gene, and *HAIKU2* (*IKU2*), a leucine-rich repeat (LRR) KINASE gene, were reported as regulators of seed size in Arabidopsis (Luo *et al.*, 2005). Mutants in either genes resulted in a reduced seed size. Although many genes regulating embryo/seed size have been identified, kinesins possessing a similar regulatory function have remained largely unknown.

Kinesins, which are microtubule (MT)-based motor proteins, hydrolyze ATP and use the derived chemical energy to drive the motility of organelles or chromosomes and have also been shown to regulate MT dynamics by acting as MT-cross-linking and translocating factors or MT depolymerases (Vicente and Wordeman *et al.*, 2015). Few kinesins functioning in the regulation of embryo/seed size have been investigated. For example, SRS3 (small and round seed), a novel kinesin 13 protein, regulates rice seed length (Kitagawa *et al.*, 2010). SGL, (short grain length), a kinesin-like protein, regulates grain length and plant height in rice (Wu *et al.*, 2014). In our previous study (Tian *et al.*, 2016), a novel member of the kinesin-12 subfamily, NtKRP, was isolated from *Nicotiana tabacum*, which was shown to be expressed in tissues with actively dividing cells and in embryos at different developmental stages. We revealed that NtKRP plays a vital role in both cell cycle progression and cell expansion in embryonic and postembryonic development. The downregulation of *NtKRP* resulted in a reduction in cell number in the embryo and a shortening in the meristematic zone of the embryonic root because of delayed cell cycle progression at the G2/M transition phase, ultimately resulting in small embryos/seeds and delayed germination of the seeds. These data indicate that NtKRP contributes to the establishment of embryo/seed size by regulating cell cycle progression. However, how NtKRP plays its role in embryo/seed development is still unclear.

Few studies have examined the interaction partners of kinesins as co-regulators of cell division. Studies in animals suggest that ribosomal proteins are candidate partners. For example, the kinesin KIF4 was shown to mediate the anterograde translocation and position of a major ribosomal constituent protein, P0, to axons (Bisbal *et al.*, 2009), suggesting that KIF4 transports this ribosomal protein to targeting sites. In addition, deletion of the 40S ribosomal protein S6 in mice was shown to block cell proliferation (Volarevic *et al.*, 2000a). In humans, the 60S ribosomal proteins RPL5 and RPL11 play essential roles in cell proliferation (Teng *et al.*, 2013). In Arabidopsis, ribosomal proteins were reported to be involved in the regulation of leaf, root, trichome, and embryo development (Byrne, 2009). To date, the evidence for plant kinesins interacting with ribosomal proteins to co-regulate cell proliferation is not yet available. Whether this is a conserved mechanism in plants and animals deserves extensive investigation.

Here, we report that NtRPL17 directly interacts with NtKRP, playing an important role in establishing the size of embryos/seeds by regulating cell cycle progression. Our findings reveal an essential role of ribosomal proteins in seed development and identify a new strategy to regulate the size of embryos/seeds.

## Materials and methods

### Plant materials

*N. tabacum* L. cv. Petite Havana SR1 plants used in this study were grown in pots at 25°C in a greenhouse under a 16 h light/8 h dark cycles. *Nicotiana benthamiana* plants were grown in pots at 21°C in a greenhouse under a 14 h light/10 h dark cycles.

### Phylogenetic analyses

Homologous sequences of NtRPL17 from different plants were obtained from the National Center for Biotechnology Information (NCBI) server using BLASTP. A multiple sequence alignment of the known ribosomal protein L17 family was conducted using CLUSTALW (www.ebi.ac.uk/Tools/msa/clustalw). Phylogenetic analysis of different RPL17s was performed with the software Molecular Evolution Genetics Analyses version (MEGA) 5.05, applying the neighbor-joining method.

### Yeast two-hybrid screening and assay

Matchmaker yeast two-hybrid vectors were purchased from Clontech. The DNA-binding domain vector pGBKT7 carries the *TRP1* nutritional marker and the activation domain vector pGADT7 carries the *LEU2* nutritional marker for selection in yeast. Yeast techniques and two-hybrid screening methods were performed according to the yeast protocols handbook and the Matchmaker GAL4 two-hybrid system manual (Clontech). NtKRP-Tail (amino acids 754–1194) was cloned in pGBKT7 vector, generating a fusion with the GAL4 DNA-binding domain, pGBKT7-NtKRP-Tail. A tobacco shoot apex and young leaf tissue cDNA library was constructed using the vector pGADT7 containing the GAL4 activation domain (AD) in host strain AH109 (Clontech). The yeast host strain Y187 (Clontech) was transformed with pGBKT7-NtKRP-Tail as a bait, and the cDNA library was screened using the yeast mating method. Cells were selected on medium lacking Leu, Trp, His, and Ade (SD/–Leu/Trp/His/Ade). About 3**×**10^6^ clones were screened for NtKRP-interacting proteins and about 1000 positive clones could grow on SD/–Leu/-Trp/-His/-Ade selective medium. Among them, 408 colonies were detected to activate a third *MEL1* reporter via adding X-ɑ-gal as a substrate, which indicates the presence of strong protein-protein interactions. The plasmids were then isolated from these colonies and digested with *Hind* III restriction enzyme. In total, 303 colonies were >500 bp and 216 positive colonies, which represent 76 genes, were obtained after re-tests. Following elimination of false positive clones, 22 positive clones encoding putative NtKRP-interacting proteins remained.

### GST pull-down assays

The tail domain (2263–3582 bp) of NtKRP and NtRPL17 (1–525 bp) were cloned into the prokaryotic expression vectors pGEX4T-1 and pET28a to generate pGEX4T-1-NtKRP-Tail and pET28a-NtRPL17, respectively. Sequence data used can be found in the NCBI GenBank (http://www.ncbi.nlm.nih.gov/Genbank) under the following accession numbers: KP100646 for *NtKRP* and KP100647 for *NtRPL17*. The GST/His-tagged fusion proteins were expressed in *E*. *coli* BL21 (DE3) cells. GST-NtKRP-Tail and His-NtRPL17 fusion proteins were incubated with 50 µL of immobilized glutathione resin at 4°C for 1 h and 2 h, respectively. The resins were then washed with 1 mL of 100 mM glutathione elution buffer (Pierce). The pull-down eluates were electrophoresed on SDS 10% polyacrylamide gels, and proteins were transferred to nitrocellulose membranes for western blotting. To detect the NtKRP-Tail protein, we used the monoclonal anti-GST antibody (Tiangen Biotech) diluted 1:10000. To detect the NtRPL17 protein, we used the monoclonal anti-His antibody (Tiangen Biotech) diluted 1:6000.

### Bimolecular fluorescence complementation (BiFC)

BiFC constructs were prepared by applying the vectors pSPYNE-35S and pSPYCE-35S for split YFP N-terminal/C-terminal fragment expression, respectively. The vectors pSPYNE-35S and pSPYCE-35S were digested with *Hind*III and *Eco*RI. Fragments containing the expression cassettes were ligated into the plant transformation vector pCAMBIA1300, generating pCAMBIA-SPYNE and pCAMBIA-SPYCE. The full-length ORF of *NtKRP* was inserted in-frame into the vector pCAMBIA-SPYCE to generate pCAMBIA-SPYCE-NtKRP. Similarly, the genes *NtRPL17* and *CDKB1-1* were cloned into pCAMBIA-SPYNE, yielding pCAMBIA-SPYNE-NtRPL17 and pCAMBIA-SPYNE-CDKB1-1. The BiFC constructs were introduced into *Agrobacterium tumefaciens* strain GV3101 via electroporation, and the resulting bacterial suspensions were delivered into *N. benthamiana* leaf cells by infiltration (Voinnet *et al.*, 2003; Würtele *et al.*, 2003). The transformed plants were kept at 21°C for 2 d. Enhanced YFP signals in leaf epidermal cells were observed using confocal microscopy (Olympus FluoView FV1000). Co-expression of NtKRP-SPYCE and CDKB1-1-SPYNE was used as a negative control. Results presented are representative of three separate infiltrations for every test.

### Gene expression analysis

Isolation of the embryos at different developmental stages was described previously (Zou *et al.*, 2004). Total RNA was extracted from roots, stems, leaves, root tips, stem tips, and flowers using the TRIzol reagent (Ambion). All total RNAs were digested with RNase-free DNase I (Promega) and cDNA was synthesized using Transcriptor reverse transcriptase (Roche) according to the manufacturer’s instructions, followed by RT-qPCR. Glyceraldehyde-3-phosphate dehydrogenase (GAPDH) was used as an internal control for RT-qPCR. All data from RT-qpcr analysis represent the mean ± standard error from three independent experiments. All primers used in this work are listed in Supplementary Table 1 at *JXB* online.

### In situ *hybridization*

The 417 bp 3’ end of *NtRPL17* was cloned into the pGEM-T Easy (Promega) vector containing T7 and SP6 polymerase and used as template. NcoI restriction enzyme was used to linearize the template. The linearized template was used for labelled RNA probe synthesis using *in vitro* transcription in the presence of digoxigenin-11-UTP (DIG). The root tips, stem tips, and ovules of tobacco were fixed with buffered 4％ paraformaldehyde solution and embedded in paraffin. The paraffin blocks were cut into 8 µm thick sections. The reaction results of *in situ* hybridization signals were detected as a dark purple color by adding the substrates nitroblue tetrazolium/5-bromo-4-chloro-3-indolyl-phosphate (NBT/BCIP) (Tian *et al.*, 2016). The hybridization signals were observed with an OLYMPUS IX71 microscope.

### Subcellular localization of NtRPL17

To investigate the subcellular distribution of NtRPL17, the full-length cDNA of *NtRPL17* was first fused in-frame with EGFP and inserted between the CaMV 35S promoter and the NOS terminator in the pUC19 vector, generating *p35S::NtRPL17*-EGFP vector. Then the construct was transfected in onion (*Allium cepa*) epidermal cells through particle-mediated transient transformation using PDS-1000/He System according to the manufacturer’s protocol (Bio-Rad). NtRPL17-GFP was expressed in onion epidermal cells. To further confirm the subcellular localization of NtRPL17, the full-length NtRPL17 expression construct *p35S::NtRPL17*-EGFP, namely GFP fused to the C terminus of NtRPL17, was transiently transferred into leaf epidermal cells of *N. benthamiana.* NtRPL17-CGFP fusion protein expression was visualized in transformed onion epidermal cells and leaf epidermal cells of *N. benthamiana* under a confocal laser scanning fluorescence microscope (Olympus FluoView FV1000, Tokyo, Japan). In addition, the full-length NtRPL17 expression construct *p35S::NtRPL17*-EGFP, namely GFP fused to the N terminus of NtRPL17, was also transiently transferred into leaf epidermal cells of *N. benthamiana*. For colocalization analysis with an ER (endoplasmic reticulum) marker, NtRPL17-GFP was co-expressed in onion epidermal cells with an ER marker containing an N-terminal signal peptide derived from an Arabidopsis vacuolar basic chitinase and the C-terminal amino acid sequence HDEL (ER-RFP) (Zhao *et al.*, 2014). Coating of gold particles and bombardment were performed according to the manufacturer’s instructions (Bio-Rad Laboratories).

### RNAi vector construction and tobacco transformation

RNAi constructs were generated using pKANNIBAL and pART27 vectors. A 258 bp region (KI: 1–258 bp) from the N-terminus and a 262 bp region (KB: 261 bp–522 bp) from the C-terminus of *NtRPL17* were respectively inserted into the *Xho*I and *Kpn*I sites in front of the *PDK* intron in the pKANNIBAL vector, the same fragments in reverse orientations were cloned between the *Cla*I and *Xba*I sites just after the intron to generate the intermediate vectors pKANNIBAL-KI and pKANNIBAL-KB. The RNAi cassettes assembled in pKANNIBAL were transferred into the binary vector pART27 as *Not*I digested fragments, yielding the final RNAi constructs pART27-KI and pART27-KB. After the sequences of the resulting constructs were confirmed, the pART27-derived vectors were introduced into *A. tumefaciens* strain GV3101 by electroporation and transformed into *N. tabacum* wild-type SR1 plants by the leaf disc method. The primers used for NtRPL17-KI/KB sense-F, NtRPL17-KI/KB sense-R, introducing *Xho*I and *Kpn*I sites, and NtRPL17-KI/KB anti-F，NtRPL17-KI/KB anti-R, introducing *Xba*I and *Cla*I sites, are listed in Supplementary Table S1.

### Seed germination and radicle growth

The tobacco seeds of wild-type and *NtRPL17* RNAi plants were surface sterilized for 1 min with 70% ethanol and for 8 min with 10% sodium hypochlorite containing 4% active chlorine. The seeds were washed three times with sterile distilled water and stratified for 48 h at 4°C. Seeds were plated on 1/2 Murashige and Skoog (MS) glass culture dishes with 1.0% (w/v) sucrose. Agar plates were placed in a greenhouse at 25°C under 16 h light/8 h dark cycles. Germination frequencies were calculated after 2 d. To draw the root growth curves, the locations of the root tips were marked daily at the same time after seed germination. At least three replicates were performed. Images were collected by scanning and analyzed using ImageJ software (http://rsb.info.nih.gov/ij/).

### Embryo and root tip analysis

The embryos and root tips were first fixed overnight in Carnoy’s fluid (ethanol:acetic acid with a 3:1 ratio, v/v) and then hydrated in a graded ethanol series (70%, 50%, 30%, 10%) and water, respectively. The hydrated embryos and root tips were transferred in Hoyer’s solution, comprising 30 g Arabic gum, 200 g chloral hydrate, and 20 g glycerine in 50 mL of water, for whole mount clearing. After 24 h, the cleared embryos and root tips were observed under an inverted phase contrast microscope (Olympus CK-30) and a Leica DMIRE 2 fluorescence microscope equipped with a cooled CCD camera (RS image MicroMAX, Princeton Instruments). The images were analyzed using ImageJ software.

### Flow cytometric analysis of nuclear DNA content

To examine cell cycle progression status, the DNA profiles of wild-type and *NtRPL17*-silenced transgenic plants root tip cells were compared by flow cytometric analysis. Root tips of about 1–2 mm were excised, immediately chilled on ice, and chopped in a cold room with a single-edged razor blade in a glass petri dish 5 cm in diameter in Galbraith’s buffer, comprising 45mm MgCl_2_, 30mm sodium citrate, 20mm 4-Morpholinepropane sulfonic acid (MOPS), and 1mg/ml Triton X-100 at pH 7.0 (Galbraith *et al.*, 1983). The cellular debris was passed through a 300 µm mesh nylon filter into a sample tube. The released nuclei in the filtrate were stained with 50 µg/mL propidium iodide in the presence of 50 µg/mL RNase. After 30 min, the stained nuclei were analyzed by flow microfluorometry with a Beckman Coulter CyAn ADP flow cytometer cell sorter operating at a laser wavelength of 488 nm and a power output of 20mw, measuring at least 10000 nuclei per sample (Dolezel *et al.*, 2007).

## Results

### Identification of ribosomal protein NtRPL17 as an NtKRP-interacting protein

**Fig. 1.**
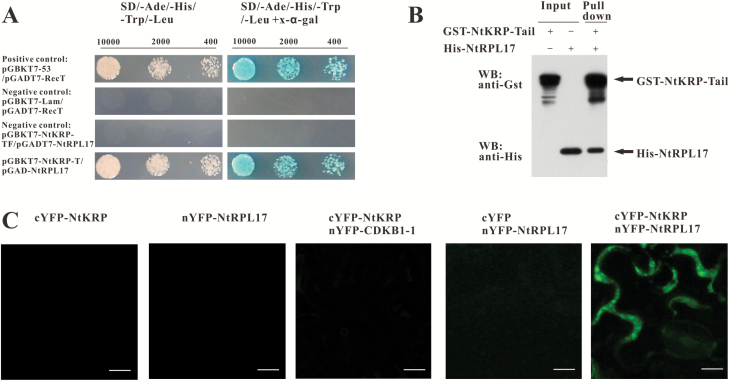
Interaction between NtRPL17 and NtKRP *in vitro* and *in vivo*. (A) Identification of NtRPL17 interaction with NtKRP via yeast two-hybrid assay. Yeast two-hybrid screening of tobacco shoot apex and young leaf cDNA library, carried out using NtKRP-Tail as bait, identified NtRPL17 as a potential protein interacting with NtKRP. High stringency selective plates lacking leucine, tryptophan, histidine, and adenine were used to grow interacting clones. Yeast α-galactosidase activity was determined in plates containing 2mg/ml X-α-gal as a chromogenic substrate. The vectors and expressed proteins are indicated. pGBKT7-P53/pGADT7-RecT serves as a positive control. pGBKT7-Lam/pGADT7-RecT and pGBKT7-NtKRP-TF/pGADT7-NtRPL17 serve as negative controls. NtKRP-TF indicates a fragment of NtKRP running from 2263–2982 bp. (B) Identification of the direct physical interaction between NtRPL17 and NtKRP by GST pull-down analysis *in vitro*. *E.coli*-expressed His-NtRPL17 was purified and incubated with purified GST-NtKRP-Tail immobilized on glutathione-Sepharose 4B beads. Bound protein was analyzed by western blotting using the anti-His antibody. GST-NtKRP-Tail protein was analyzed using the anti-GST antibody. (C) BiFC analysis of the interaction between NtRPL17 and NtKRP in tobacco epidermal cells. Note that fluorescence was only detected when nYFP-NtRPL17 and cYFP-NtKRP (full length) were co-expressed. NtKRP was located in the cytoplasm and NtRPL17 was both in the cytoplasm and nucleus. CDKB1-1 is a nuclear and cytoplasmic protein (Boruc *et al.*, 2010; Tian *et al.*, 2016). SPYCE-NtKRP, SPYNE-NtRPL17, co-expression combinations of SPYCE-NtKRP+SPYNE-CDKB1-1 and SPYNE-NtRPL17+the empty vector SPYCE were used as negative controls. SPYNE and SPYCE indicate split YFP N-terminal/C-terminal fragment expression, respectively. Scale bar, 10µm.

Previous studies have shown that NtKRP plays a critical role in establishing the size of embryos/seeds by regulating cell cycle progression. To obtain further insights into the function of NtKRP, a GAL4 yeast two-hybrid screen was performed to identify NtKRP-interacting proteins. Firstly, the yeast strains AH109 and Y187 were verified as possessing the expected phenotypes. Then, the tail polypeptide of NtKRP, namely amino acids 754–1194, was fused with the pGBKT7 vector to generate pGBKT7-NtKRP-T and used as a bait protein. The pGBKT7-NtKRP-T fusion was tested for transcription auto-activation and toxicity (Supplementary Fig. S1A, B). Next, the pGBKT7-NtKRP-T fusion was used to screen a stem apex and young leaf cdna library. A total of about 3**×**10^6^ library clones were screened and 22 positive clones encoding putative NtKRP-interacting proteins were identified (Supplementary Table S2). Then, we sequenced the positive clones and assessed the cDNA sequences using the BLAST (Basic Local Alignment Tool) program. Interestingly, two 60S ribosomal proteins were identified as NtKRP-interacting proteins. The impetus to perform the yeast two-hybrid screen with NtKRP as bait protein was to identify candidate binding partners that may point to a role for cooperating with NtKRP in regulating cell division. Recent evidence has actually shown that ribosomal proteins play roles in regulating cell division (Volarevic *et al.*, 2000b; Kim *et al.*, 2004; Wang *et al.*, 2006; Badhai *et al.*, 2009; Wu *et al.*, 2011; Li *et al.*, 2012; Wang *et al.*, 2014; Xu *et al.*, 2015; Yang *et al.*, 2016). We therefore chose a ribosomal protein for further analysis. This protein repeated 20 times in the cDNA library and is a potential NtKRP-interacting target protein (Fig. 1A). Analysis of its ORF using the program ORF Finder from NCBI indicated that the gene contained a complete ORF of 525 nucleotides which encoded a protein of 175 amino acids. A BLASTP (Protein Basic Local Alignment Tool) search in NCBI GenBank databases revealed that the gene encoded a 60S ribosomal large subunit protein L17 in *N. tabacum*. Thus, the gene was named *NtRPL17*.

To further confirm the specific interaction between NtRPL17 with NtKRP, we performed a glutathione S-transferase (GST) pull-down assay by first expressing the N-terminal tail domain NtKRP-Tail:GST tagged protein and an NtRPL17:His tagged protein in *Escherichia coli* BL21. As seen in Fig. 1B, the GST-Tail specifically bound His-NtRPL17 when the former served as bait. The result provided additional evidence of a specific and direct physical interaction between NtKRP and NtRPL17 *in vitro*.

To further examine the interaction between NtRPL17 and NtKRP *in planta*, we also performed a BiFC assay. NtKRP-Tail and NtRPL17 were expressed as fusions with the C- and N- terminal portions of yellow fluorescent protein, cYFP and nYFP. *A. tumefaciens* cells expressing both *NtKRP-Tail-cYFP* and *NtRPL17-nYFP* were co-infiltrated into leaves of *N. benthamiana*. A positive interaction was indicated by the reconstitution of YFP, with the resulting fluorescence detectable by confocal microscopy. The results showed that NtKRP-Tail indeed interacted with NtRPL17 *in vivo* and that the NtRPL17/NtKRP complex was localized to the cytoplasm of the co-transformed epidermal cells of *N. benthamiana* (Fig. 1C). This result is consistent with a physical interaction between NtKRP and NtRPL17. In addition, the interaction specifically occurred in the cytoplasm, consistent with our previous observation of the subcellular localization of NtKRP.

Taken together, we conclude that NtKRP specifically and directly interacts with NtRPL17, a 60S ribosomal large subunit protein. The two proteins are likely to be involved in the same pathway regulating both embryo development and radicle growth.

### Protein sequence and phylogenetic analysis of NtRPL17

Multiple sequence alignment revealed that NtRPL17 is highly similar to RPL17s from *Arabidopsis thaliana*. The highly conserved region, belonging to the ribosomal protein L17 family signature, was found in the C-terminus. Moreover, several conserved sites also showed that NtRPL17 was classified as a ribosomal protein L17 family member (Supplementay Fig. S2A). In order to investigate the evolutionary relationship among different RPL17s and RPL17-like proteins from different species, a phylogenetic tree of RPL17s was constructed using the neighbor-joining method. Phylogenetic analysis showed NtRPL17 is most closely related to RPL17 proteins from *Nicotiana tomentosiformis* (Supplementary Fig. S2B). NtRPL17 and RPL17-1 from *N. tomentosiformis* are separately located in a small clade of the phylogenetic tree, consistent with a distinct role for this ribosomal protein in plant development.

### NtRPL17 is predominantly expressed in meristematic tissues and embryos

**Fig. 2.**
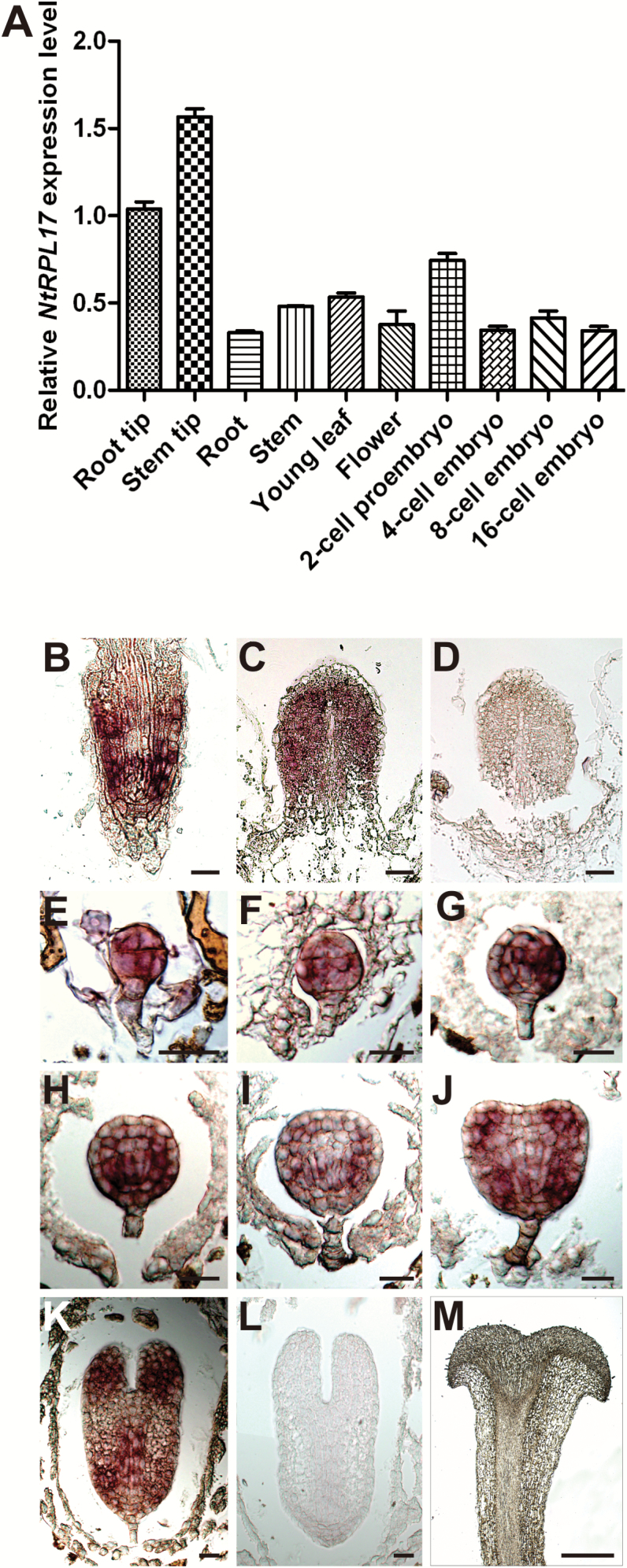
Expression pattern of NtRPL17. (A) RT-qPCR analysis of *NtRPL17* in both vegetative tissues and flower and in the embryos from two-celled proembryos to 16-celled embryos. The expression level of *NtRPL17* in the root tip was set to 1. (B–M) RNA *in situ* hybridization of *NtRPL17* in wild-type plants, showing a longitudinal section of root meristem (B), stem meristem (C), stem meristem probed with a sense probe as a negative control (D), 16-celled embryo (E), 32-celled embryo (F), 64-celled embryo (G), small globular embryo (H), globular embryo (I), heart-shaped embryo (J), torpedo-shaped embryo (K), torpedo-shaped embryo probed with a sense probe as negative control (L) and stigma (M). Scale bars, 50µm (B–D), 25 µm (E–L), 500 µm (M).

*NtRPL17* tissue expression profiles were investigated using total RNA extracted from root tip, shoot tip, 2-celled proembryos, 4-, 8-, and 16-celled embryos, root, stem, and young leaves via RT-qPCR (quantitative real-time reverse transcription PCR). The results of RT-qPCR showed a high level of expression of *NtRPL17* in meristematic tissues, including the root tip, shoot apex tissues, and embryos at different developmental stages (Fig. 2A).

In order to accurately examine *NtRPL17* tissue expression patterns, *in situ* hybridization was also applied. Detailed analysis indicated that *NtRPL17* was strongly expressed in root meristem, stem meristem, and embryos at different developmental stages. All these tissues have plenty of dividing cells. However, no signal was detected in the mature pistil (Fig. 2B–M). This expression pattern revealed by *in situ* hybridization is consistent with RT-qPCR data.

### NtRPL17 is localized in the cytoplasm and nucleus

**Fig. 3.**
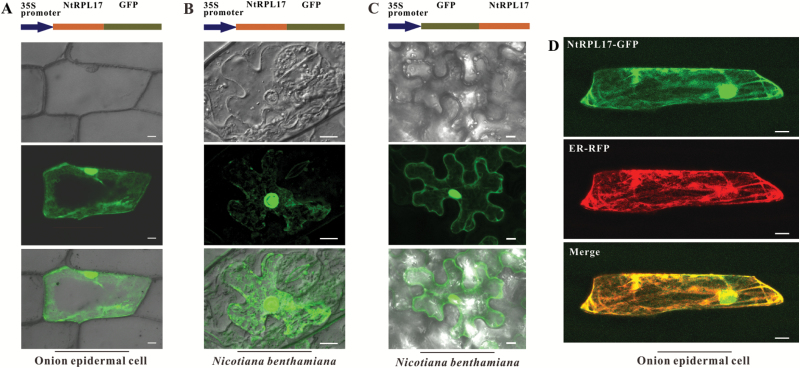
Subcellular localization of NtRPL17. (A, B) The epidermal cells of onion (A) and *N. benthamiana* leaves (B) were transiently transfected with Agrobacterium strain GV3101 harboring plasmids expressing of *pro35S::NtRPL17-GFP*. NtRPL17-CGFP, namely GFP fused to the C terminus of NtRPL17, exhibited distribution in both the cytoplasm and the nucleus. (C) The leaf epidermal cells of *N. benthamiana* were transiently transfected with Agrobacterium strain GV3101 harboring plasmids expressing of *pro35S::NtRPL17-GFP*. NGFP-NtRPL17, namely GFP fused to the N terminus of NtRPL17, exhibited distribution in both the cytoplasm and the nucleus. (D) NtRPL17 showed partial co-localization with the ER marker ER-RFP. Samples were collected 2 d after infiltration and visualized by confocal laser scanning microscopy. Onion: *n*=9, *N. benthamiana*: *n*=12. Scale bars, 20µm in (A), 10µm in (B) and (C), 30µm in (D).

We examined the intracellular localization of NtRPL17. To determine the subcellular localization of NtRPL17, we transiently expressed the C-terminal GFP fusion construct of *NtRPL17* driven by the 35S promoter in onion (*A. cepa*) and *N. benthamiana* leaf epidermal cells. Confocal microscopy detection of the GFP fluorescence revealed that NtRPL17-CGFP is localized in both the cytoplasm and the nucleus. We also transiently expressed the N-terminal GFP fusion construct of *NtRPL17* driven by the 35S promoter in leaf epidermal cells of *N. benthamiana*. NGFP-NtRPL17 also localized to the cytoplasm and nucleus, exhibiting a highly similar distribution to the C terminal NtRPL17-CGFP fusion protein (Fig. 3A–C). To test whether NtRPL17 is co-localized with ER, we performed a co-localization assay with an ER marker in the epidermal cells of onion. The results showed that NtRPL17 partially co-localized with the ER marker (Fig. 3D).

### Downregulation of NtRPL17 results in embryonic root growth defects

A previous report revealed that downregulation of *NtKRP* mainly resulted in the retardation of embryonic root growth via a reduction in cell numbers in the meristematic zone and alterations in cell elongation in the differentiation zone (Tian *et al.*, 2016). In this report we also confirmed via screening the yeast two-hybrid cDNA library that NtRPL17 is an interacting protein of NtKRP. We therefore further hypothesized that NtKRP-NtRPL17 could cooperatively affect embryonic root development after seed germination. To explicitly address this hypothesis, we constructed two independent RNAi plasmids (Supplementary Fig. S3A, B) to downregulate *NtRPL17* expression and confirmed its significant downregulation using RT-qPCR (Fig. S4A). Two transgenic lines of the two RNAi plants with obvious downregulated expression levels of *NtRPL17*, K17I-10 and K17B-(11), were used to determine the function of NtRPL17. The confirmed non-transgenic lines and wild-type SR1, with normal *NtRPL17* expression, served as the controls.

**Fig. 4.**
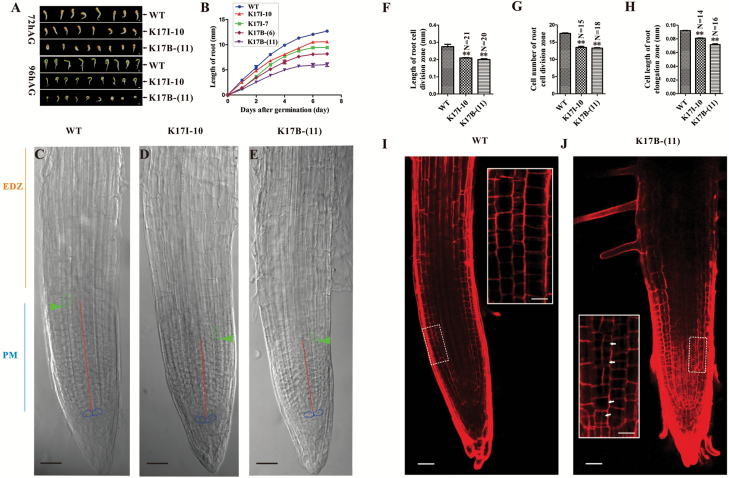
Down-regulation of *NtRPL17* resulted in embryonic root growth defects after seed germination. (A) Seeds 72 h and 96 h after germination (AG) of wild-type plants and K17I-10 and K17B-(11) RNAi plants. Scale bar, 100µm (72 h AG), 0.5 mm (96 h AG). (B) Root growth curves of wild-type and RNAi transgenic plants. Values are means ± standard deviation, *n*≥20. (C–E) Root tip of wild-type plants (C), K17I-10 (D) and K17B-(11) (E) RNAi plants at 4 days post-germination (dpg). PM, proximal meristem; EDZ, elongation and differentiation zone. Green arrowheads indicate the first elongated cortex cell (cortex transition zone). The red lines mark the length of the meristematic zone. The blue dotted lines marked the quiescent center. Scale bar, 10µm in (C–E). (F) Length of the root meristematic zone of wild-type and RNAi plants. Values are means ± standard deviation, *n*≥20. (G) Cell number of the root meristematic zone of wild-type and RNAi plants. Values are means ± standard deviation, *n*≥15. (H) Cell length in the root elongation zone of wild-type and RNAi plants. Values are the means ± standard deviation, *n*≥14. Double asterisk indicates statistical difference compared with wild-type, Student’s *t*-test, *P*<0.01, in (F), (G) and (H). (I, J) The abnormal cell division orientation in NtRPL17 RNAi root tip. (I) The division pattern and morphology of root tip meristematic cells in the wild-type. (J) The cell division orientations and cell shapes of root tip meristem in *NtRPL17* RNAi transgenic plants. The white arrows in (J) indicate the cells resulting from irregular cell division. Scale bars, 50µm in (I) and (J).

As downregulation of *NtKRP* induced slower embryonic root development by reducing cell numbers in the meristematic zone and alterations in cell elongation in the differentiation zone (Tian *et al.*, 2016), we followed the same developmental process in the two *NtRPL17* RNAi transgenic lines. We found that all planted seeds ultimately germinated, but the transgenic seeds required more time than the control plants. Thus, the germination velocity of seeds from the two transgenic lines, but not their germination rate, was significantly reduced compared with the wild-type control (Supplementary Fig. S4B). Moreover, significant differences in plant height were observed between the RNAi lines and the controls when they developed into complete plants (Supplementary Fig. S4C). The differences were proportional to the level of *NtRPL17* downregulation (Supplementary Fig. S4D). To further investigate the retardation of plant development by *NtRPL17* deficiency, we measured the second, third, fourth, and fifth internodes from the bottom of the plants (Supplementary Fig. S4E). The result showed that the downregulation of *NtRPL17* caused shorter internodes (Supplementary Fig. S4F). In addition, the root length of the germinated seed of the RNAi plant was significantly shorter than that of the corresponding control (Fig. 4A). The much slower development of the *NtRPL17*-silenced transgenic lines was evident from the growth curves of the roots after germination (Fig. 4B) and the shorter lengths of the meristematic zones of the root tip (Fig. 4C–F). In addition, the number of cells in the meristematic zones was significantly reduced (Fig. 4G), whereas no difference was observed in the structure of the quiescent center. An analysis of cell length in the root elongation zones showed significant differences between transgenic and control plants (Fig. 4H).

In addition, the living root tip meristematic cells were stained with 10µg/ml propidium iodide in order to observethe cell division pattern. Detailed observation revealed that the root tip meristematic cells of *NtRPL17* RNAi transgenic plants presented irregular cell division patterns and irregular cell shapes compared with that of wild-type root tips (Fig. 4I, J). The results implied that the downregulation of *NtRPL17* not only resulted in the slowdown of cell division but also irregularity in cell division patterns.

Collectively, these data provided evidence that the downregulation of *NtRPL17* hinders embryonic root growth after seed germination by reducing both the number of cells in meristematic zones and cell expansion in differentiation zones, which was similar to the observation in the *NtKRP* RNAi lines.

### Downregulation of NtRPL17 causes smaller embryos and seeds

**Fig. 5.**
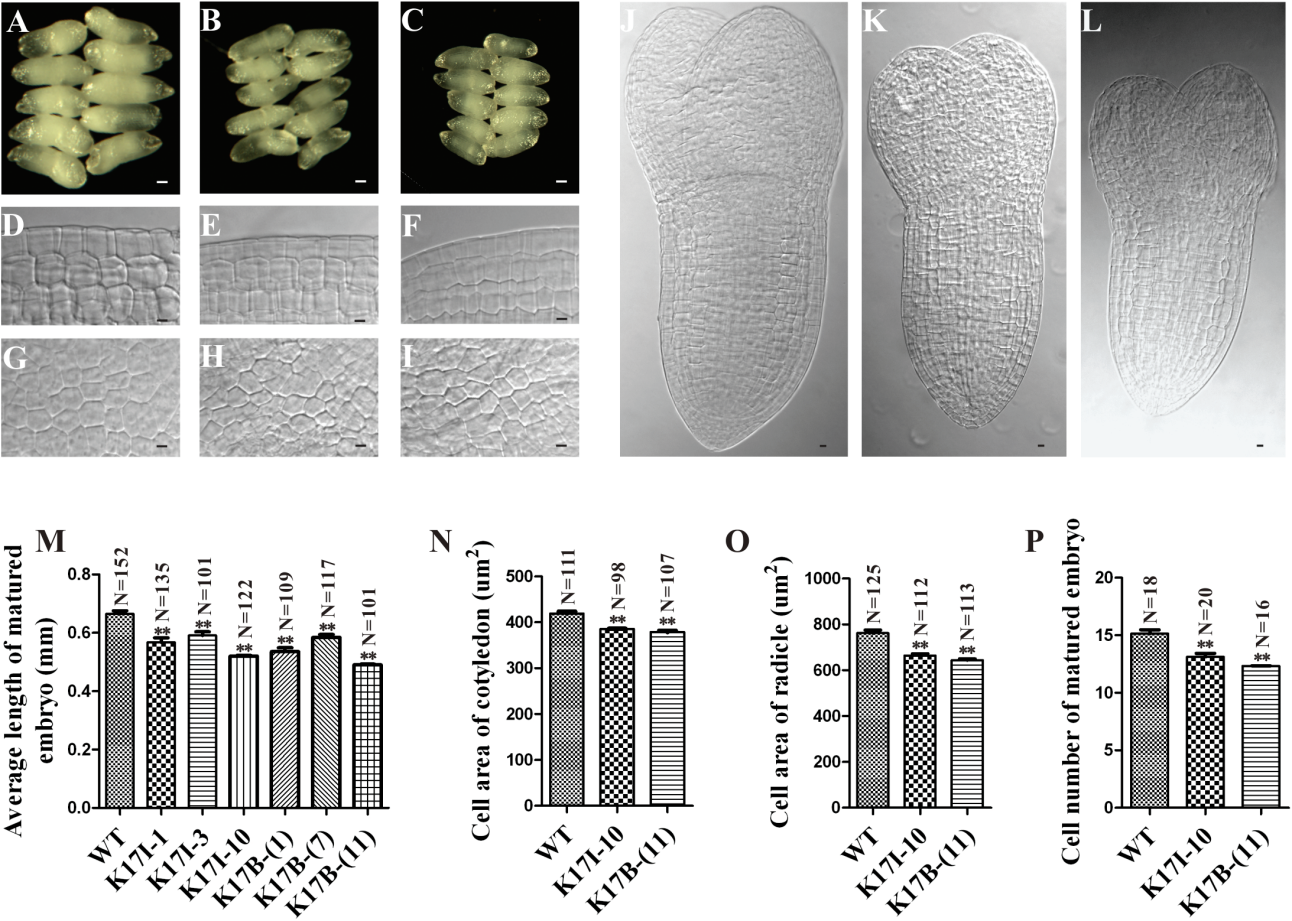
Phenotypic characterization of wild-type and *NtRPL17* RNAi embryos. (A–C) Comparison of mature embryo morphologies for wild-type (A), *NtRPL17* RNAi line K17I-10 (B) and *NtRPL17* RNAi line K17B-(11) (C). (D–F) Longitudinal views of radicle cells in the matured embryos of wild-type (D), *NtRPL17* RNAi plant K17I-10 (E) and K17B-(11) (F). (G–I) Longitudinal views of cotyledon cells in the matured embryos of wild-type (G), *NtRPL17* RNAi plant K17I-10 (H) and K17B-(11) (I). (J–L) The matured embryos of wild-type (J), *NtRPL17* RNAi line K17I-10 (K) and K17B-(11) (L), displaying the cell number difference among them. (M) Length of matured embryos of wild-type and *NtRPL17* RNAi plants. Values are means ± standard deviation, *n*≥101. Cell area analysis of cotyledon (N) and radicle (O) of wild-type and RNAi plants. Values are means ± standard deviation, *n*≥98 and *n*≥112, respectively. (P) Cell number in the radicles of wild-type and *NtRPL17* RNAi plants. Values are means ± standard deviation, *n*≥16. Scale bars, 100 µm in (A–C), 10 µm in (D–L). Double asterisk indicates significant difference compared with wild-type, Student’s *t*-test, *P*<0.01, in (M), (N), (O) and (P). (This figure is available in colour at *JXB* online).

**Fig. 6.**
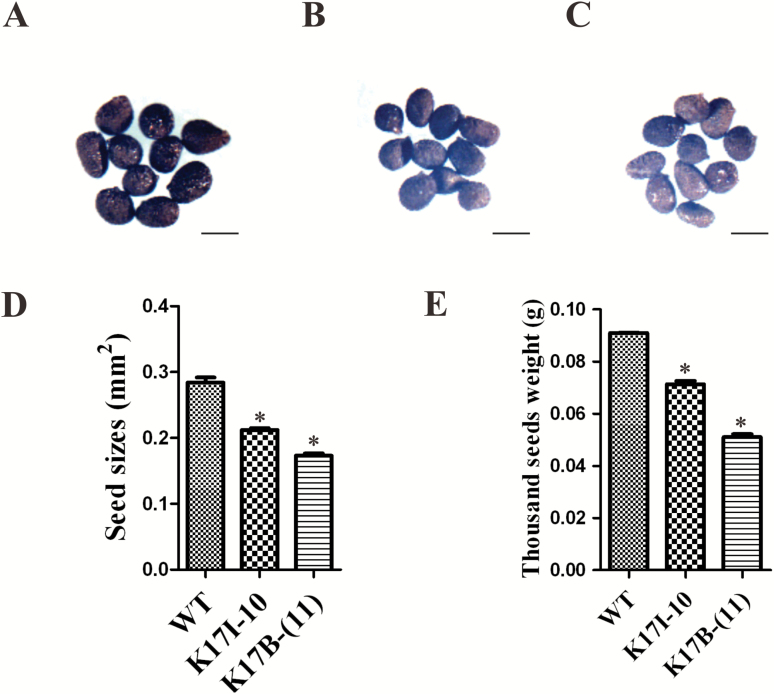
The seed sizes in the wild type (SRI) and *NtRPL17* RNAi transgenic lines. Seed size comparison among wild type SRI (A), *NtRPL17* RNAi transgenic line K17I-10 (B), and NtRPL17 RNAi transgenic line K17B-(11) (C). (D) Seed size comparison between the wild type (SRI) and *NtRPL17* RNAi transgenic lines. Values are means ± standard deviation, *n*≥106. (E) The thousand-seed weight in the wild type (SRI) and *NtRPL17* RNAi transgenic lines. Values are means ± standard deviation, *n*=1000. Asterisks indicate signiﬁcant differences with respect to the wild-type, Student’s *t*-test *P*<0.05. Scale bars, 0.5 mm in (A–C).

To address the role of NtRPL17 in embryogenesis, we examined the embryonic development of *NtRPL17* RNAi lines K17I-10 and K17B-(11), respectively. A comparison of mature embryos between those isolated from control and *NtRPL17* RNAi transgenic K17 I -10 or K17B-(11) lines showed that embryos from *NtRPL17* RNAi lines were much smaller (Fig. 5A–C and 5J–L), although the plants retained their ability to develop into mature embryos with normal structures. In the cotyledon and radicle of fixed, cleared embryos (Fig. 5D–I, M), significant differences between the control and transgenic plants were determined both in the cell areas (Fig. 5N, O) and in cell numbers (Fig. 5P). Thus, the decreased size of embryos from *NtRPL17* RNAi transgenic plants was due not only to a reduction in cell number, but also due to a reduction in cell size, which again suggests that NtRPL17, together with NtKRP, is involved in regulating the cell cycle and cell expansion. Furthermore, an examination of seed size and thousand-grain weight of *NtRPL17* RNAi transgenic plants showed that the seeds were significantly smaller than wild-type SR1 seeds (Fig. 6A–D) and also significantly lighter (Fig. 6E).

In summary, these results demonstrated that the downregulation of *NtRPL17* altered embryonic development by reducing embryo and seed sizes and therefore also seed yields. This phenotype is similar to that of *NtKRP* RNAi transgenic plants in which *NtKRP* was downregulated and provides further support for NtRPL17 and NtKRP as partners in the same regulatory pathway.

### NtRPL17 is required for the G2/M phase transition

**Fig. 7.**
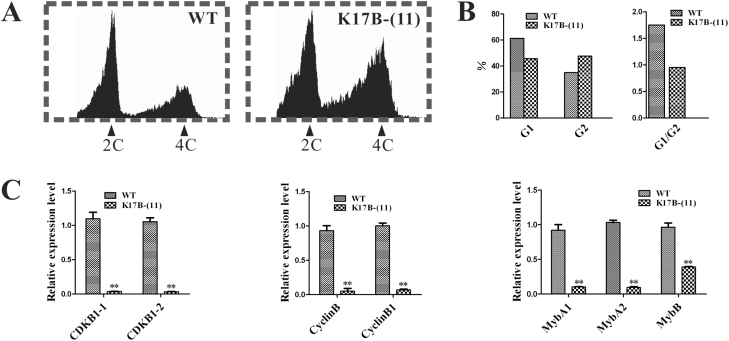
Cell cycle progression is hindered at the G2/M transition in *NtRPL17*-silenced lines. (A) The DNA profiles of propidium iodide (PI)-stained root tip nuclei of wild-type and *NtRPL17*-silenced lines as measured in a flow cytometer. (B) Quantification of the DNA profiles in (A). (C) Relative expression levels of G2/M phase-specific genes in *NtRPL17*-silenced line and wild-type root tips. Double asterisks indicate significant difference from the wild-type, Student’s *t*-test *P*<0.01.

The above phenotype analysis of *NtRPL17* RNAi plants provided a clue that NtRPL17 might also be a cell cycle progression-related protein, similar to its interacting protein NtKRP. To investigate whether *NtRPL17* is expressed in tissues with active cell division, we constructed a *proNtRPL17::GFP* plasmid and transferred it into wild-type plants. The results showed that the GFP driven by the native *NtRPL17* promoter was abundantly expressed in meristematic tissues of the root tip, which contains a large number of dividing cells (Supplementary Figure S5), suggesting its possible role in cell division. Next the DNA profiles of root tip nuclei from the wild-type and the two transgenic lines were examined using flow cytometry to define whether the specific cell cycle phase was also affected in *NtRPL17*-silenced lines. As shown in Fig. 7, fewer cells were in G1 phase because most of them were in G2 in RNAi transgenic plants, resulting in a lower G1/G2 ratio than that in wild-type (Fig. 7A, B), presumably because the cells were blocked to a certain extent at the G2/M transition stage.

It is well known that the basic regulatory machinery governing the cell cycle and the G2/M transition relies on cyclin dependent kinase (CDK) complexes and transcription factors. We therefore used RT-qPCR to examine the expression levels of cyclin genes (CYC), CDK genes, *NtmybA1*, and *NtmybA2* in RNAi transgenic and wild-type seedlings as we previously reported. NtmybA1 and NtmybA2 encode Myb-like transcription factors (Araki *et al.*, 2004) and are expressed specifically at the G2/M phase. Both genes have roles in the G2/M phase by modulating the expression of B-type cyclin genes and may regulate a suite of co-expressed genes. The results revealed that two CYC genes, two CDK genes, and two Myb-like transcription factors were downregulated by 25–50% in the two RNAi transgenic lines compared with the control. These data suggest that the expression of G2/M-specific genes is downregulated in *NtRPL17*-silenced transgenic lines and thus NtRPL17 is likely required for the G2/M transition by maintaining the normal function of these G2/M-specific genes.

## Discussion

In angiosperms, a mature seed is usually composed of embryo, endosperm, and seed coat, which are respectively derived from zygote, fertilized central cell, and maternal integuments. Several signaling pathways that determine seed size by influencing cell proliferation and cell expansion in maternal tissues or endosperm have been identified in Arabidopsis and rice. These signaling pathways include the IKU (HAIKU) pathway, including a VQ motif protein HAIKU1, IKU1, a leucine-rich repeat kinase, IKU2, a WRKY transcription factor, MINI3, and a transcriptional coactivator, SHB1, and integrating cytokinin and abscisic acid signaling, the ubiquitin-proteasome pathway, G-protein signaling, MAPK signaling, phytohormones and transcriptional regulatory factors (Li and Li, 2016). These works reveal a complex regulatory network for seed size control and also indicate that much more detailed investigations should be carried out into embryo development at the level of cell division, cell growth, and cell differentiation to elucidate the mechanism of establishing seed structure and morphology.

A previous study indicated that NtKRP regulates embryo/seed size and embryonic root growth via regulating cell cycle progression at the G2/M transition. To further investigate the molecular mechanism of NtKRP function, we constructed a yeast two-hybrid cDNA library and screened for NtKRP-interacting proteins. NtRPL17 was identified as an NtKRP-interacting protein by screening the cDNA library. Sequence and phylogenetic analyses shows that NtRPL17 encodes a ribosomal protein that is a component of the 60S large subunit. Ribosomal proteins aren’t the only constituent elements of simple translation machines and ribosomes, and impart a role in ribosome biogenesis and post-translational modifications of proteins. Research increasingly shows that many ribosomal proteins could perform additional extra-ribosomal functions, independent of protein biosynthesis, in the regulation of diverse cellular processes. Recent advance studies have shown that many ribosomal proteins, including the proteins of ribosomal large and small subunits, regulate cell proliferation via influencing cell cycle progression. Quite a few ribosomal proteins, including S6, S15A, S19, S24, S27A, L6, L15, L26, L29, L34, and L36A, were reported to promote cell proliferation (Volarevic *et al.*, 2000b; Kim *et al.*, 2004; Wang *et al.*, 2006; Badhai *et al.*, 2009; Wu *et al.*, 2011; Li *et al.*, 2012; Wang *et al.*, 2014; Xu *et al.*, 2015; Yang *et al.*, 2016). For example, the knockdown of RPS19 or RPS24 in primary fibroblast cells from Diamond-Blackfan anemia (DBA) patients triggered cell cycle arrested at the G1 phase and G2/M phase, respectively, leading to a marked reduction in cell proliferation capacity (Badhai *et al.*, 2009). Similarly, RPL6 was overexpressed in human gastric cancer tissues and its overexpression could accelerate cell growth through promoting the G1 to S phase transition (Gou *et al.*, 2010). Silencing of RPL6 in human gastric cancer cells suppressed cell proliferation via inhibiting cell cycle progression at the G1 to S phase transition (Wu *et al.*, 2011). In plants, some ribosomal proteins have been reported to directly regulate the cell division. In the vegetative tissues of Arabidopsis, loss of function of the ribosomal proteins SHORT VALVE1 (STV1), POINTED FIRSTLEAF2 (PFL2), PIGGY-BACK1 (PGY1), PIGGYBACK2 (PGY2), PIGGYBACK3/ASYMMETRIC LEAVES1/2 ENHANCER6/OLIGOCEL-LULA5 (PGY3/AE6/OLI5), ASYMMETRIC LEAVES 1/2 ENHANCER (AE5), and OLIGOCELLULA7 (OLI7) reduced cell division and led to a reduction in the number of palisade mesophyll cells (Ito *et al.*, 2000; Nishimura *et al.*, 2005; Pinon *et al.*, 2008; Yao *et al.*, 2008; Fujikura *et al.*, 2009). The mitochondrial ribosomal L18 protein HES plays important roles in cell division and seed development by regulating cell cycle progression (Zhang *et al.*, 2015). These works revealed that some ribosomal proteins may play an unusual role in some specific developmental processes. The data presented in this work suggest that NtRPL17 is one of these proteins.

In barley, RPL17 was abundantly expressed in dividing tissues, such as the young leaves and root tip, implying that RPL17 may promote cell division (Madsen *et al.*, 1991). In tobacco, RPL17 is most strongly expressed when the rate of cell division is highest in some tissues and much more weakly expressed when the tissues are mature (Gao *et al.*, 1993). This implies that RPL17 might play a role in regulating cell division. In the insect *Apis cerana cerana*, AccRPL17 is mainly localized in young developing tissues in larvae and embryos (Meng *et al.*, 2010), implying that AccRPL17 might also play an important role in insect embryo development by regulating cell division. In this work, using RT-qPCR and *in situ* hybridization, we showed that NtRPL17 is preferentially expressed in tissues where cell division is active, including the young leaves, root tip, stem tip, and developing embryos. Furthermore, we confirmed that RPL17 participated in regulating embryo/seed development through influencing cell cycle progression and cell expansion in plants, showing a similar mechanism as that in animals. This suggests a conserved expression pattern and a basic regulatory role of RPL17 in cell proliferation and cell expansion in embryo development.

According to our investigations, the expression profiles of *NtRPL17* exhibit an overlap with that of *NtKRP* in most plant tissues. The detection of subcellular localization of NtRPL17 showed that NtRPL17 is localized in both the cytoplasm and nucleus. In general, the gene expression pattern and protein subcellular localization of NtRPL17 are overlapping, but not exactly the same as that of NtKRP. Notably, the phenotypes of *NtRPL17*-silenced lines were similar to those of *NtKRP*-silenced lines, with respect to delayed cell cycle progression and reduced cell numbers in the embryonic root. NtKRP and NtRPL17 therefore seem to act as partners required for cell cycle progression in specific tissues and jointly regulate the G2/M transition, thus modulating the size of embryos/seeds. These data revealed a previously unidentified link between a kinesin and a ribosomal protein that is critical for seed development in plants. This finding may also lead to new approaches to investigate the molecular mechanisms regulating cell division. In animal studies, kinesins have been shown to transport ribosomal proteins to targeting sites and to regulate cell cycle progression (Volarevic *et al.*, 2000a). This might also be the story in plants. In this case, our work provides a valuable springboard for further investigations.

## Supplementary Data

Supplementary data are available at *JXB* online.

Figure S1. Detection of pGBKT7-NtKRP/Tail fusion for transcription auto-activation and toxicity.

Figure S2. Sequence alignment and phylogenetic analysis of NtRPL17 protein.

Figure S3. Schematic maps for constructions of the RNAi intermediate vectors pKANNIBAL-NtRPL17-KI/KB and final vectors pART27- NtRPL17-KI/KB.

Figure S4. Down-regulation of *NtRPL17* resulted in plant development retardation.

Figure S5. GFP expression under the *NtRPL17* promoter is abundant in root tip meristematic tissues.

Supplementary Table S1. The primer sequences used in this study.

Supplementary Table S2. Identified potential NtKRP-interacting proteins from yeast two-hybrid cDNA library screening.

## Supplementary Material

Supplementary Material

## Abbreviations:

BiFC: bimolecular fluorescence complementation
ER: endoplasmic reticulum
MT: microtubule
RT-qPCR: quantitative real-time reverse transcription PCR.
