# Supplementary material for: Ribosomal protein NtRPL17 interacts with kinesin-12 family protein NtKRP and functions in the regulation of embryo/seed size and radicle growth
Source: J Exp Bot. 2017 Oct 14;68(20):5553–64. doi: 10.1093/jxb/erx361 (PMC5853406; doi:10.1093/jxb/erx361)
Supplement: Supplementary Material [file erx361_suppl_supplementary_material.pdf]

**Ribosomal protein NtRPL17 interacts with kinesin-12 family protein NtKRP and functions in the regulation of embryo/seed size and radicle growth**

Shujuan Tian[1], Jingjing Wu[1], Yuan Liu[1], Xiaorong Huang[1], Fen Li[1,2], Zhaodan Wang[1] and Meng-Xiang Sun[1**]

1. Department of Cell and Developmental Biology, College of Life Sciences, State Key Laboratory of Hybrid Rice, Wuhan University, Wuhan, China, 430072
2. College of Life Sciences, Henan Normal University, Xinxiang, China, 453007

**SUPPLEMENTARY DATA**

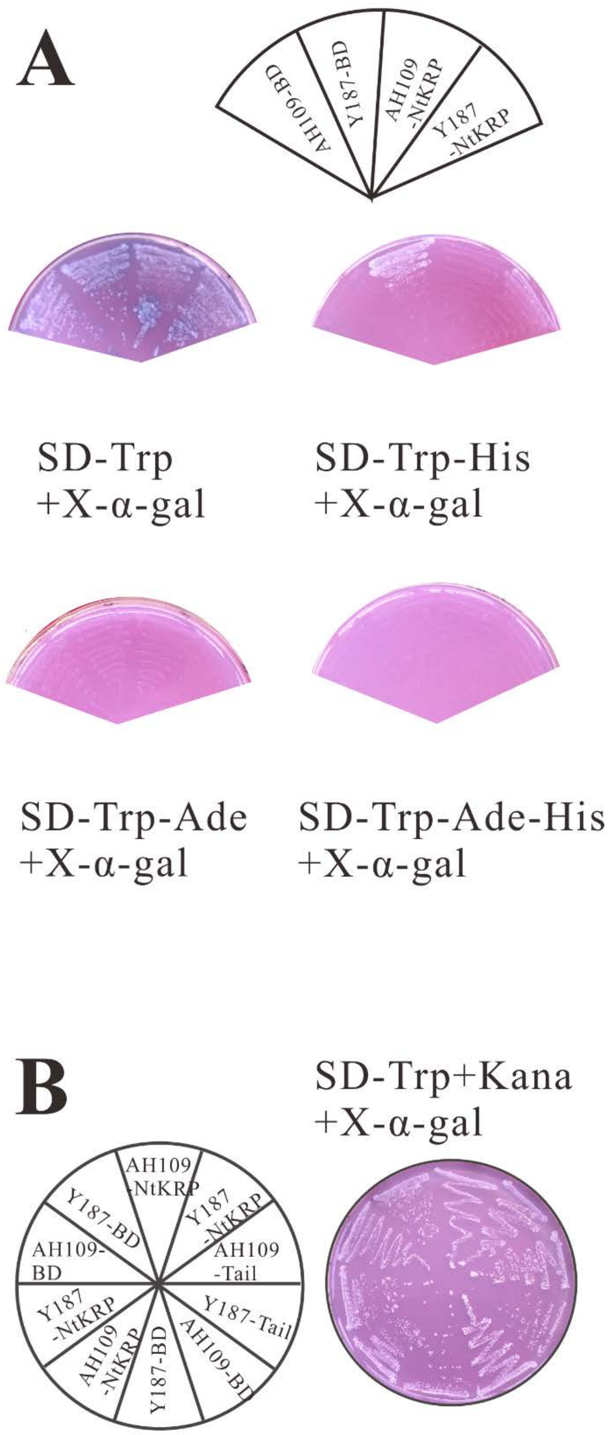


**Supplementary Figure S1. Detection of pGBKT7-NtKRP/Tail fusion for transcription auto-activation and toxicity.** (A) The test of pGBKT7-NtKRP fusion for transcriptional auto-activation. pGBKT7-NtKRP fusion plasmid was transformed in yeast strains AH109 and Y187. pGBKT7 empty plasmid was used as control. The transformants were respectively placed on SD/-Trp, SD/-Trp-His, SD/-Trp-Ade and SD/-Trp/-Ade/-His selective mediums containing X-α-gal. The Y187 transformant

colonies could not grow on SD/-Trp-His, SD/-Trp-Ade and SD/-Trp/-Ade/-His mediums, which indicates that there was no leaked expression of *ADE2 and HIS3* genes in the Y187 strains. However, AH109 transformants exhibited background growth on SD/-Trp-His medium and could not grow on SD/-Trp-Ade and SD/-Trp/-Ade/-His mediums. So the selective mediums QDO SD/-Ade/-His/-Leu/-Trp medium could be used for the library screening. (B) The test of pGBKT7-NtKRP/Tail fusion for toxicity. Individual pGBKT7, pGBKT7-NtKRP and pGBKT7-NtKRP-Tail BD plasmids were transformed in yeast strains AH109 and Y187. One large (2-3mm) colony is inoculated into 50ml of SD/-Trp/+Kana (20μg/ml) liquid culture and incubated at 30℃ overnight with shaking at 250rpm. The $OD_{600}$ of the culture was checked and is >0.8. Therefore, pGBKT7-NtKRP and pGBKT7-NtKRP-Tail fusion were non-toxicity for yeast strains AH109 and Y187 and could be used to construct and screen the two-hybrid library. Kana= kanamycin.

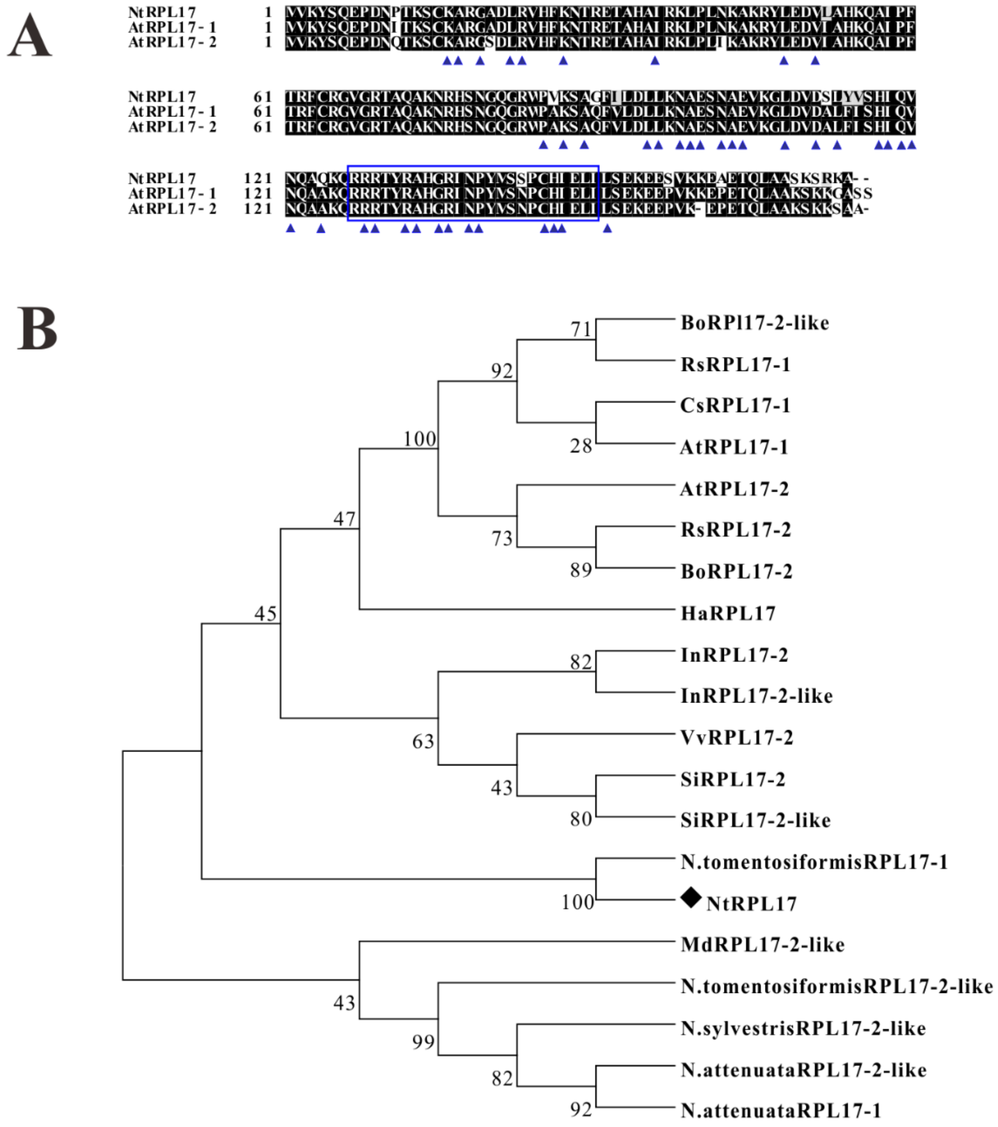


**Supplementary Figure S2. Sequence alignment and phylogenetic analysis of NtRPL17 protein.** (A) Alignment of NtRPL17 with related members of large 60S ribosomal subunit protein L17, including *Arabidopsis thaliana* AtRPL17-1 (GenBank accession number: AT1G27400) and *Arabidopsis thaliana* AtRPL17-2 (AT1G67430). The short sequence motif that was characteristic of ribosomal protein L17 family is boxed, and RPL17s conserved sites are marked by (▲). (B) A bootstrap consensus of phylogenetic tree representing similarities of NtRPL17 protein sequence with those of *Arabidopsis thaliana* AtRPL17-1 (GenBank accession number: AT1G27400), *Arabidopsis thaliana* AtRPL17-2 (AT1G67430), *Brassica oleracea* BoRPL17-1-like (XP_013626728.1), *Brassica oleracea* BoRPL17-2 (XP_013592363.1), *Camelina*

*sativa* CsRPL17-1 (XP_010499218.1), *Helianthus annuus* HaRPL17 (OTG05323.1), *Ipomoea nil* InRPL17-2 (XP_019151617.1), *Ipomoea nil* InRPL17-2-like (XP_019164091.1), *Malus domestica* MdRPL17-2-like (XP_008379114.1), *Nicotiana attenuata* RPL17-1 (OIT20857.1), *Nicotiana attenuate* RPL17-2-like (XP_019239646.1), *Nicotiana sylvestris* RPL17-2-like (XP_009803259.1), *Nicotiana tabacum* NtRPL17 (KP100647), *Nicotiana tomentosiformis* RPL17-1 (XP_009631407.1), *Nicotiana tomentosiformis* RPL17-2-like (XP_009591574.1), *Raphanus sativus* RsRPL17-1 (XP_018450554.1), *Raphanus sativus* RsRPL17-2 (XP_018442884.1), *Sesamum indicum* SiRPL17-2 (XP_011071217.1), *Sesamum indicum* SiRPL17-2-like (XP_011086770.1) and *Vitis vinifera* VvRPL17-2 (XP_002279293.1). The tree was calculated with MEGA 5.05 software using neighbor joining method. RPL17, ribosomal protein L17.

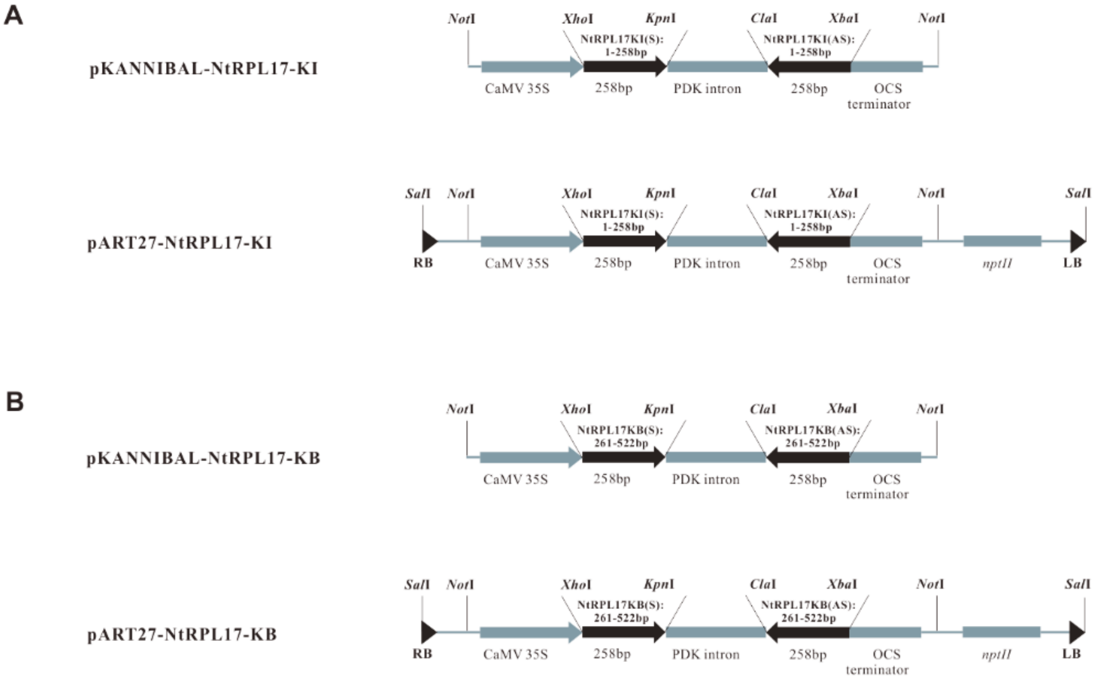


**Supplementary Figure S3. Schematic maps for constructions of the RNAi intermediate vectors pKANNIBAL-NtRPL17-KI/KB and final vectors pART27- NtRPL17-KI/KB.** (A) Sketch map of the RNAi intermediate vector pKANNIBAL-NtRPL17-KI and final vector pART27- NtRPL17-KI. NtRPL17-KI(S): sense insertion of the RNAi target fragment of *NtRPL17* gene (1-258bp). NtRPL17-KI (AS): anti-sense insertion of the same fragment as “S”. (B) Sketch map of the RNAi intermediate vector pKANNIBAL-NtRPL17-KB and final vector pART27- NtRPL17-KB. NtRPL17-KB(S): sense insertion of the RNAi target fragment of *NtRPL17* gene (261bp-522bp). NtRPL17-KB (AS): anti-sense insertion of the same fragment as “S”.

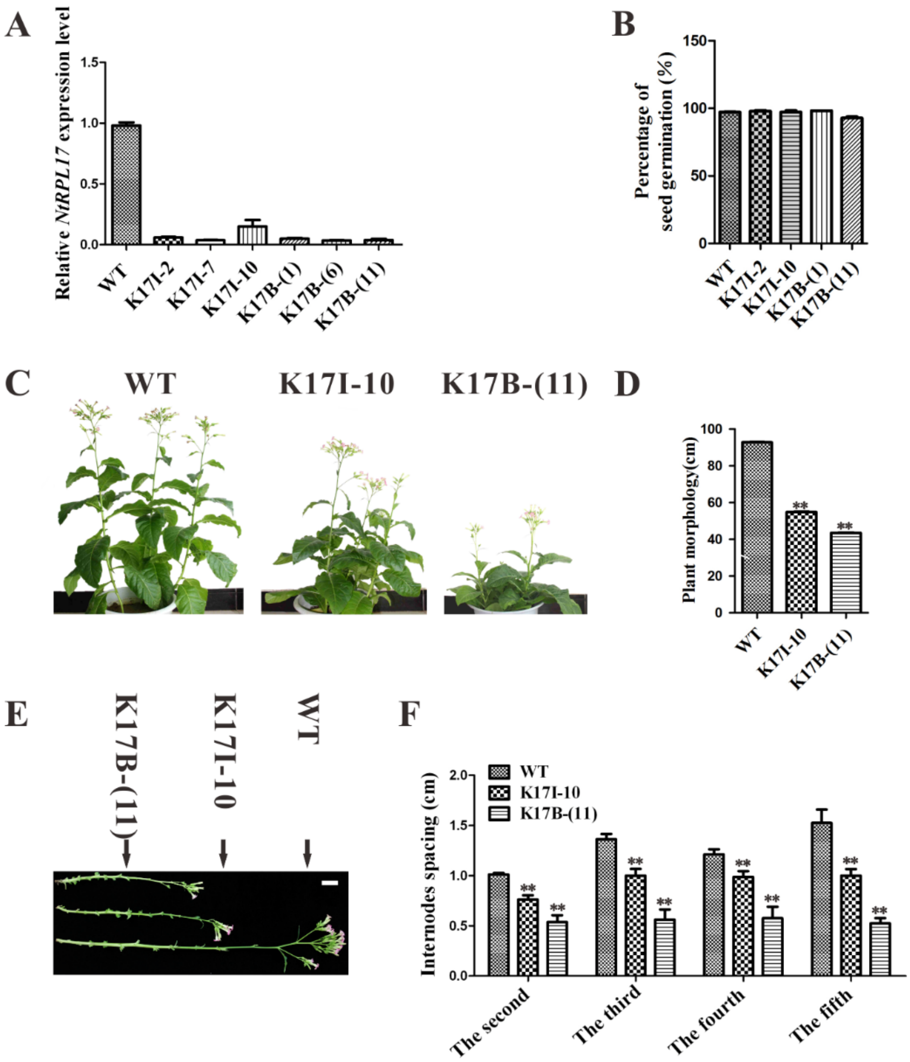


**Supplementary Figure S4. Down-regulation of *NtRPL17* resulted in plant development retardation.** (A) Relative expression levels of *NtRPL17* in RNAi transgenic and wild type seedlings measure by RT-qPCR. The expression level of *NtRPL17* in the wide type (WT) is set to 1. (B) Seed germination percentage of wild type and *NtRPL17* RNAi transgenic plants. (C) The plant morphological comparison between wild-type and RNAi plants. (D) The plant height statistics analysis of wild type and *NtRPL17* RNAi transgenic lines. Values are means ± SD (n = 20). (E) The internode spacing comparison between wild-type and RNAi transgenic plant. The number of internode spacing begins from the stem bottom of plants. Scale bar =5cm. (F) The length analysis of the internode spacing starting from the stem bottom of wild-type and RNAi transgenic lines. Values are means ± SD (n = 20). Double asterisk indicates statistical difference compared to WT (t-test, $p<0.01$) in (D) and (F).

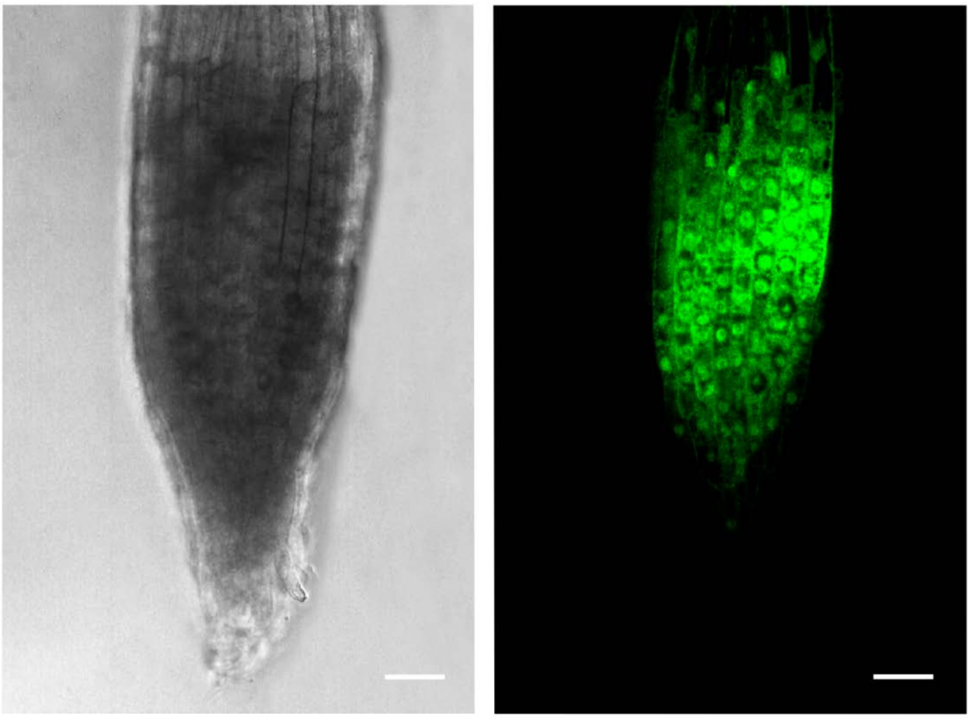

**Supplementary Figure S5. GFP expression under *NtRPL17* promoter is abundant in root tip meristematic tissues.** Note the fluorescent signal distributed in the meristem zone. Scale bar=50μm.

**Supplementary Table S1. The primer sequences used in this study**

| Name | Forward Primer | Reverse Primer | Size (bp) |
|---|---|---|---|
| *35S* | TCTCAGAGCAGAATCG GGTAT | AGGGTCTTGCGAAGG ATAG | 1250 |
| *EGFP* | GGTGAGCAAGGGCGA GG | ACTTGTACAGCTCGTC CATG | 724 |
| *NtRPL17* | ATGGTGAAGTACTCCC AA GAGCCTGAT | CTATGCCTTCCTAGAT TT GCTTGCTGC | 525 |
| *NtRPL17-KI sense* | CTCGAGATGGTGAAGT ACTCCCAAGAGCCTGA T | GGTACCGACTGGCCA ACGTCCTTGACC | 258 |
| *NtRPL17-KI antisense* | TCTAGAATGGTGAAGT ACTCCCAAGAGCCTGA T | ATCGATACTGGCCAA CGTCCTTGACC | 258 |
| *NtRPL17-KB sense* | CTCGAGGTCTGCTGGA TTTATTCT | GGTACCTGCCTTCCTA GATTTGCTTGCTGC | 262 |
| *NtRPL17-KB-antisense* | TCTAGAGTCTGCTGGA TTTATTCT | ATCGATTGCCTTCCTA GATTTGCTTGCTGC | 262 |
| *qGAPDH* | AGGCTGGAGAAAGAA GCTACCTA | AGTCTGTGGACACCA CATCATCT | 115 |
| *qCDKB1-1* | AGTGGACAAAGAGAA GGGCATAC | AGATCCCAACAAGAC TTCAGGAG | 133 |
| *qCDKB1-2* | AGTGGACAAAGAGAA GGGCATAC | AGATCCCAACAAGAC TTCAGGAG | 133 |
| *qCyclinB* | CGTTACCTCGCTGTGA CAACTAC | ACACAAAGTCATTCA CCTCAGGA | 118 |
| *qCyclinB1* | GCCTAAAGCAGCTCAG ATTTCTC | CCTTCTTCTTGGCTGG TACATCT | 134 |
| *qMybA1* | ATGGTAAAACAGCATC CTGGAGT | GCAGCTTCAAGTCGTC TAGCATA | 149 |
| *qMybA2* | TACTATGAGCCTCCTC GTTTTCC | ATCCCACAGCCTAAAT GGAGTAA | 159 |

**Supplementary Table S2. Identified potential NtKRP-interacting proteins from yeast two-hybrid cDNA library screening**

| Code number | Accession number of homologous protein | Homologous protein | Frequency of occurrence |
|---|---|---|---|
| B20-1 | ABB87114.1 | cytochrome c oxidase family protein-like | 1 |
| C3-2 | AAF72099.1 | Pto-responsive gene 1 protein | 1 |
| C24-2 | NP_172363.19 | Histone H2A, putative | 54 |
| D3-1 | NP_188905.1 | rRNA processing protein-related | 1 |
| E8-3 | AAG35472.1 | Sulfur | 2 |
| E13-1 | P08927 | RuBisCO large subunit-binding protein subunit | 1 |
| F7-1 | ABI95860.1 | Methionine synthase | 2 |
| F22-1 | ABO07415.1 | SMC1 | 1 |
| H16-1 | BAD97359.1 | PsbQ | 20 |
| H24-2 | Q41229 | Photosystem I reaction center subunit IV B | 1 |
| J32-1 | NP_195570.1 | AtFP6 (Farnesylated protein 6) | 1 |
| K17-2 | XP_009631407 | 60S ribosomal protein L17 | 20 |
| K28-2 | AAP22954.1 | Potyvirus VPg interacting protein | 1 |
| L30-1 | AAD32567.1 | NT3 | 2 |
| P28-1 | AAZ20285.1 | ubiquitin fusion protein | 4 |
| Q26-2 | AAO34706.1 | ethylene response factor 4 | 1 |
| R10-1 | BAC53936.1 | chromomethylase-like protein | 1 |
| T16-2 | AAA34366.1 | ribosomal protein L41 | 12 |
| V36-1 | NP_186795.1 | MBD9 (Methyl-CPG-Binding Domain 9); DNA binding protein | 1 |
| W15-1 | EAW58916.1 | nuclear receptor subfamily 4, group A, member 3 | 1 |
| W27-1 | NP_565396.1 | GCIP-interacting family protein | 1 |
| X4-1 | P32980 | ATP synthase delta chain | 3 |
